# Gene expression profiling reveals aryl hydrocarbon receptor as a possible target for photobiomodulation when using blue light

**DOI:** 10.1038/srep33847

**Published:** 2016-09-27

**Authors:** Anja Becker, Anna Klapczynski, Natalia Kuch, Fabiola Arpino, Katja Simon-Keller, Carolina De La Torre, Carsten Sticht, Frank A. van Abeelen, Gerrit Oversluizen, Norbert Gretz

**Affiliations:** 1 Medical Research Centre, University of Heidelberg, D-68167 Mannheim, Germany; 2 Philips Group Innovation, Research, High Tech Campus 34, 7.031, 5656 AE Eindhoven, The Netherlands

## Abstract

Photobiomodulation (PBM) with blue light induces a biphasic dose response curve in proliferation of immortalized human keratinocytes (HaCaT), with a maximum anti-proliferative effect reached with 30min (41.4 J/cm2). The aim of this study was to test the photobiomodulatory effect of 41.4 J/cm2 blue light irradiation on ROS production, apoptosis and gene expression at different time points after irradiation of HaCaT cells *in vitro* and assess its safety. ROS concentration was increased 30 min after irradiation. However, already 1 h after irradiation, cells were able to reduce ROS and balance the concentration to a normal level. The sudden increase in ROS did not damage the cells, which was demonstrated with FACS analysis where HaCaT cells did not show any sign of apoptosis after blue light irradiation. Furthermore, a time course could be seen in gene expression analysis after blue light, with an early response of stimulated genes already 1 h after blue light irradiation, leading to the discovery of the aryl hydrocarbon receptor as possible target for blue light irradiation.

The skin serves as a protective barrier between the internal milieu and the environment. Its outer layer, the epidermis, consists mainly of keratinocytes, which form the cornified layer comprising cross-linked proteins (cornified cell envelope) and lipids (cornified lipid envelope) and are most affected by external stimuli^1^. Besides structural scaffolding, keratinocytes actively produce substances like cytokines, neurotransmitters and hormones^2^ when exposed to external stimuli like temperature, pressure, pain, and light^3^.

Light is connected to various functions of the human body like vitamin-D metabolism, circadian rhythm and the psychosocial state and consequently is important for human health. Phototherapy (UV), photodynamic therapy (PDT) and skin rejuvenation as well as high power surgical lasers in ophthalmology, dermatology and oncology are treatment paradigms which are already used in clinics^4^,^5^. Low level light/laser Therapy (LLLT) with non-thermal, low power visible and near-infrared light is a less prominent therapeutic application which is used to stimulate wound healing, tissue regeneration and hair growth^6^,^7^,^8^ or to reduce inflammation and alleviate pain^7^,^9^,^10^,^11^,^12^. Blue light in particular is used for different medical treatments like psoriasis^13^, neonatal jaundice^14^ and back pain^15^ and it is known to have anti-microbial^16^, anti-inflammatory^17^ and anti-proliferative effects^18^,^19^.

As LLLT is not clearly characterized the new term of photobiomodulation (PBM) was established, which is defined as: “a form of light therapy that utilizes non-ionizing forms of light sources, including lasers, LEDs, and broadband light, in the visible and infrared spectrum. It is a non-thermal process involving endogenous chromophores eliciting photophysical (i.e., linear and nonlinear) and photochemical events at various biological scales. This process results in beneficial therapeutic outcomes including but not limited to alleviation of pain or inflammation, immunomodulation, and promotion of wound healing and tissue regeneration”^4^. However, defining an effective dose for a clinical use of PBM is still a critical point as the parameters of wavelength, irradiance, fluence and delivery protocol have to be clearly defined to achieve a specific biological scenario^20^. An important point to consider when creating a PBM protocol is its biphasic dose response (Arndt-Schulz curve). Beneficial therapeutic effects can be induced with low doses of light whereas higher doses are harmful and therefore phototoxic leading to a need of defining a threshold for clinical use of PBM^12^. Although many reports describe the effectiveness of light, little is known about the mechanisms transducing the light induced signals from target molecules over downstream processes and/or gene expression to the biological effects^21^ with additional difficulty of being hardly able to differentiate between primary and secondary effects.

Proliferation of HaCaT cells after PBM with blue light revealed the well-known biphasic response curve, with a slight increase of proliferation for 7.5 min and an anti-proliferative effect for 15 min (20.7 J/cm^2^). Longer irradiation times (up to 120 min, 165.6 J/cm2) did not result in a higher anti- proliferative effect^22^.

For this study an irradiation time of 30 min (41.35 J/cm^2^) was chosen for testing the blue light effect on cells. With that it was intended to have a maximum anti-proliferative effect of blue light irradiation and in addition the lowest probability to harm the cells, respectively induce cytotoxicity. To assess the safety and identify possible target genes for PBM using blue light we performed a comprehensive gene expression analysis using Affymetrix GeneChips for the time points 1 h, 3 h and 24 h after 41.4 J/cm^2^ irradiation. A verification of selected genes was conducted with qPCR. Moreover, H_2_O_2_ concentration was tested to confirm a light induced ROS production and FACS analysis for cell apoptosis was performed as safety measurement to demonstrate that ROS production does not induce apoptosis, hence, does not harm the cells.

## Results

### Blue light increases H_2_O_2_ concentration in HaCaT cells immediately after irradiation

**Figure 1:**
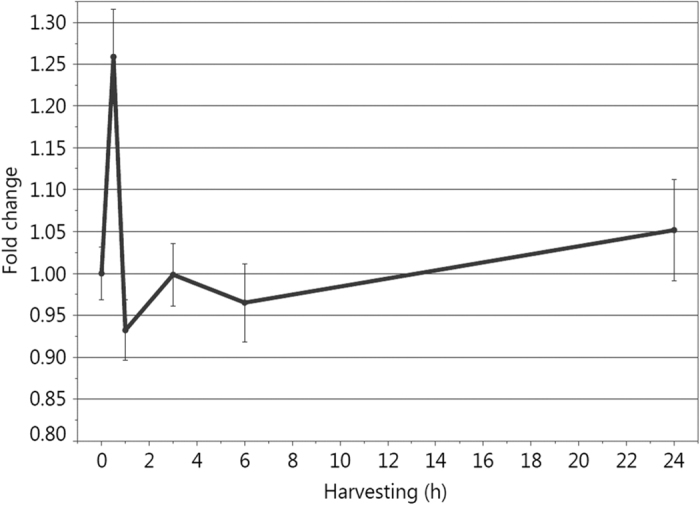
ROS measurement – blue light induces a rapid increase of H_2_O_2_ in human keratinocytes, which is balanced out by the cells within 24 h.

As light is known to induce production of ROS, respectively H_2_O_2_, we measured H_2_O_2_ concentrations in HaCaT cells at different time points after 30 min of blue light irradiation, with a first time point at 30 min according to incubation time. H_2_O_2_ concentration was increased 1.26 fold (by 26%) 30 min after blue light irradiation (p < 0.0001*). Followed by a decrease of 7% 1 h (p < 0.0001*) after irradiation, H_2_O_2_ concentration alternated between a decrease of 1% after 3 h (p = 0.7585) to 4% after 6 h (p < 0.0001*) and finally increase of 5% after 24 h (p < 0.0001*) (Fig. 1).

### Blue light irradiation does not induce apoptosis in HaCaT cells

**Figure 2:**
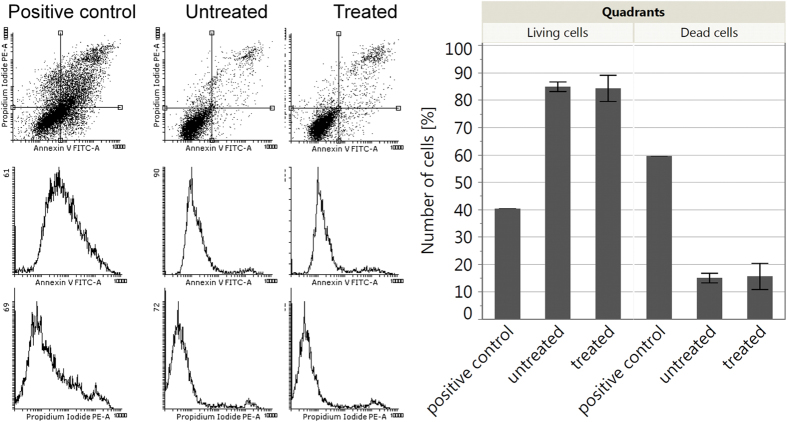
FACS analysis 24 h after 30 min of blue light irradiation. The four quadrants can be distinguished as follows: lower left quadrant = intact cells, lower right quadrant = early apoptosis, upper right quadrant = late apoptotic or secondary necrotic apoptotic cells and upper left quadrant = primary necrotic cells. For comparison between live and dead cells the lower left quadrant was used for the numbers of intact cells and the other three quadrants were taken together to show the amount of dead cells. In this graph there is no difference between early or late apoptosis or necrosis. 30 min of blue light did not induce apoptosis in HaCaT cells.

Fluorescence-activated cell sorting (FACS) was applied to test a possible apoptotic effect of blue light on HaCaT cells 24 h after 30 min irradiation. Cells were labeled with Annexin V, which binds to the phospholipid membrane component phosphatidylserine on the cell surface during early apoptosis and propidiumiodide which intercalates with DNA and therefore shows late apoptosis and cell necrosis. Staurosporine treated cells served as a positive control for induced apoptosis resulting in 40% living cells and 60% dead cells. Both untreated and light-treated cells exhibited a significant difference to the positive control (p < 0.0001). Untreated as well as blue light treated cells contained ~85% living cells and ~15% dead cells. Thus, that dose of blue light did not induce apoptosis in HaCaT cells (Fig. 2).

### Gene expression analysis reveals time course of photobiomodulatory blue light effect

Already 1 h after irradiation a change in gene expression can be observed. However, differentially regulated genes increase in number with increasing harvesting time after blue light irradiation from 1358 genes after 1 h, to 1686 genes after 3 h, to 2192 genes after 24 h (Table 1 and Supplementary data 4).

**Figure 3:**
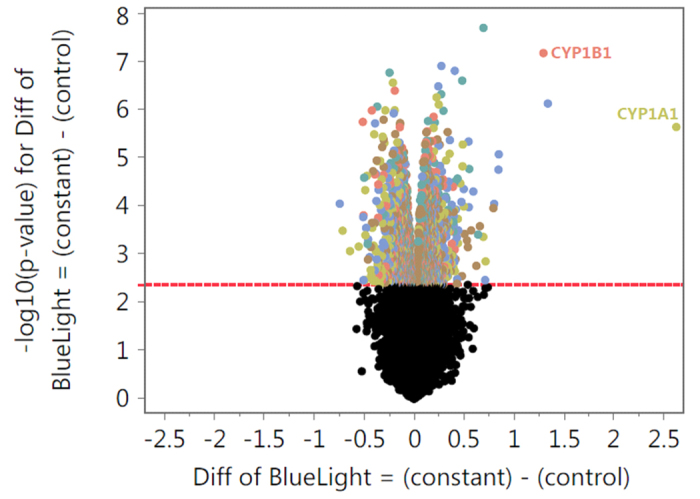
Gene expression analysis-volcano plot 24 h after blue light irradiation.

Genes that stand out are cytochrome P450 family 1 subfamily A member 1 (CYP1A1) and CYP1B1, which are both highly upregulated for all three harvesting time points, with significant p-values for 3 h and 24 h (Fig. 3 and Supplementary data 1-2).

**Figure 4:**
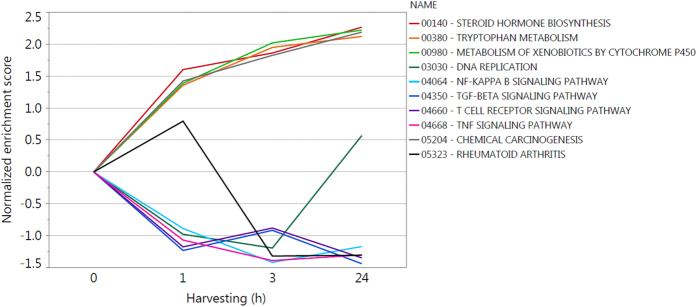
Gene set enrichment analysis-time course of selected pathways for further evaluation of gene expression.

In a next step, gene set enrichment analysis (GSEA) was performed using Kyoto Encyclopedia of Genes and Genomes (KEGG) database (Table 1). Pathways containing the highest number of deregulated genes are depicted in Fig. 4. Already 1 h after blue light irradiation genes connected to steroid hormone biosynthesis, metabolism of xenobiotics by cytochrome P450, chemical carcinogenesis and tryptophan metabolism are upregulated. The number of genes and intensity of upregulation increases for all these pathways with time from 1 h to 3 h and 24 h after irradiation. On the other hand, pathways containing downregulated genes that are reduced already 1 h after irradiation are processes like NF-κB signaling pathway, TNF signaling pathway, T cell receptor signaling pathway and TGF-β signaling pathway. These pathways are mainly linked to inflammation and infection. For NF-κB signaling pathway and TNF signaling pathway, downregulation increases from 1 h to 3 h, whereas it slightly decreases for T cell receptor signaling pathway and TGF-β signaling pathway. Nevertheless, 24 h after irradiation downregulation is higher for all these pathways when compared to 1 h after irradiation. Although rheumatoid arthritis is slightly upregulated 1 h after irradiation, the pathway is significantly downregulated 3 h and 24 h after irradiation. DNA replication is downregulated for 1 h and 3 h after blue light irradiation. Interestingly, 24 h after irradiation DNA replication is slightly upregulated.

### Gene expression analysis reveals upregulation of aryl hydrocarbon receptor target genes

**Figure 5:**
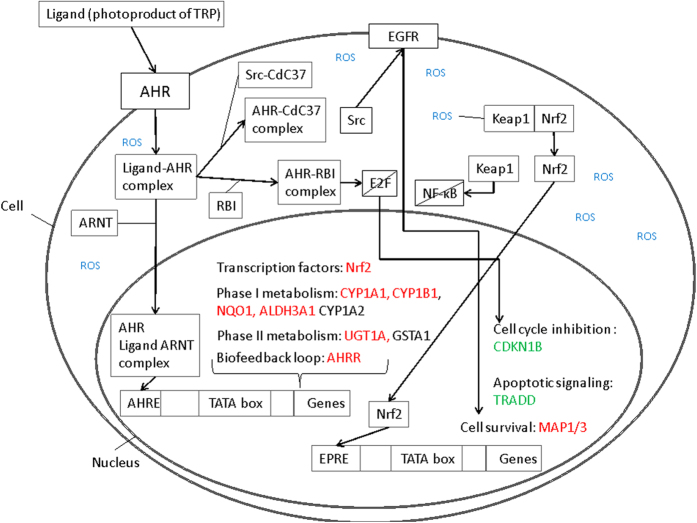
Aryl Hydrocarbon Receptor (AHR) signaling pathway. Red: upregulated gene expression after AHR activation, green: downregulated gene expression after AHR activation.

**Figure 6:**
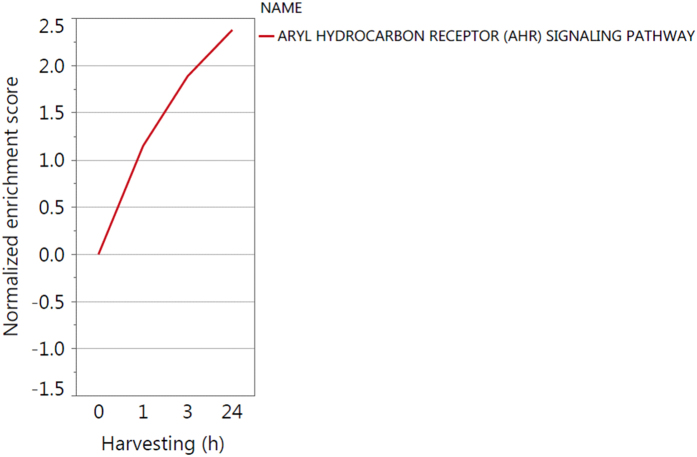
Gene set enrichment analysis-time course of the Aryl Hydrocarbon Receptor (AHR) signaling pathway.

**Figure 7:**
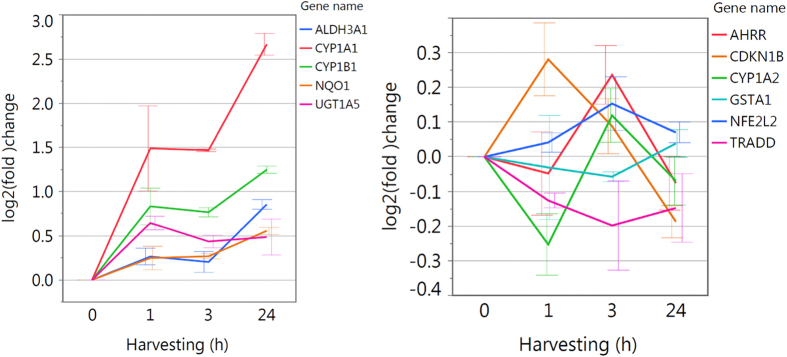
Gene expression analysis-time course of selected AHR inducible genes for further evaluation of gene expression.

CYP1A1 and CYP1B1 were both highly upregulated for all three harvesting time points leading to the identification of a possible functionality of their transcription factor aryl hydrocarbon receptor (AHR) after blue light irradiation. Tryptophan metabolism and chemical carcinogenesis are both pathways containing significantly upregulated genes and are connected to AHR. As KEGG does not provide an AHR pathway was designed (Fig. 5) using a literature search containing inter alia the “AHR battery genes” CYP1A1, CYP1A2, CYP1B1, aldehyde dehydrogenase 3 family member a1 (ALDH3A1), NAD(P)H quinone oxidoreductase 1 (NQO1), UDP glucuronosyltransferase 1 family, polypeptide A (UGT1A), glutathione S-transferase 1 (GSTA1) and genes encoding AHR and its contributors aryl hydrocarbon receptor nuclear translocator (ARNT) and aryl hydrocarbon receptor repressor (AHRR). Additionally, genes deregulated downstream after AHR activation like cyclin-dependent kinase inhibitor 1B (CDKN1B, also KIP1), nuclear factor erythroid 2 like 2 (Nrf2, also NFE2L2) and tumor necrosis factor (TNF-a) receptor-associated protein (TRADD) are depicted. The AHR signaling pathway (Fig. 6) is upregulated for all three time points with p = 0.3300 after 1 h, p < 0.0001 after 3 h and p < 0.0001 24 h after blue light irradiation. The time course of gene expression analysis for these mentioned genes is illustrated in Fig. 7 to help describing that AHR is a possible target for blue light irradiation as explained in the discussion.

CYP1A1, CYP1B1, ALDH3A1, NQO1 and UGT1A5 are upregulation already 1 h after blue light irradiation, which is stable up to 3 h. CYP1A1, CYP1B1 ALDH3A1 and NQO1 show an even higher upregulation in gene expression 24h after irradiation, whereas UGT1A keeps the same level (Fig. 7). AHRR is considerably upregulated 3 h after blue light irradiation, while CDKN1B is upregulated after 1 h and 3 h, but downregulated 24 h after irradiation. CYP1A2 is alternating from downregulation after 1 h to upregulation after 3 h and not regulated after 24 h. GSTA1 is not considerably regulated, whereas NFE2L2 (Nrf2) is upregulated after 3 h and 24 h. TRADD is downregulated for all three time points with a maximum after 3 h.

### Real time PCR verifies gene expression analysis.

To confirm microarray results genes were selected for real time PCR (qPCR). Criteria for selection were pathways with high normalized enrichment scores (NES) and/or fold changes of specific genes and connection to AHR signaling pathway. qPCRs were performed with RNA samples from harvesting time 24 h after 30 min of blue light irradiation, which were beforehand used for gene expression analysis. qPCR results match with the previously obtained gene expression results with CYP1A1, CYP1B1, ALDH3A1, NQO1 and UGT1A being significantly upregulated and FBJ murine osteosarcoma viral oncogene homolog (FOS), interleukin 8 (IL8) and keratin 5 (Krt5) being significantly downregulated (Supplementary data 3).

## Discussion

Photobiomodulatory effects of blue light irradiation on human keratinocytes were tested with functional experiments for ROS concentrations and apoptosis detection for safety issues. Furthermore, a comprehensive evaluation of gene expression analysis for the time points 1 h, 3 h and 24 h after 30 min (41.4 J/cm^2^) of blue light irradiation was conducted, which revealed a time course of gene expression and the AHR as a possible target for PBM with blue light via photo-oxidation of tryptophan.

H_2_O_2_ concentrations were tested in cells to confirm a light induced ROS production. ROS levels were increased 1.26 fold 30 min after irradiation (Fig. 1). Interestingly, the cells could balance that rise already after 1 h and concentrations alternated between slightly increased and decreased until 24 h after irradiation. These results fit to the phenomenon called mitohormesis, which is the adaptive response of mitochondria to varying ROS levels. In general, ROS, which are produced mainly in mitochondria, are signaling molecules induced by stress and an increased demand for readily available energy, which triggers the retrograde response; a process causing in transcriptional changes in the nucleus^23^,^24^. In more detail, ROS oxidize e.g. thiol groups on cysteine residues thereby activating downstream processes by changing functions of the enzymes in a signaling pathway^25^ leading to a reversible signal transduction mechanism^26^. They are able to precondition the organism thereby inducing cellular defense mechanisms that finally serve as a long-term protective shield^27^ and even prevent cellular damage^23^,^24^. Furthermore, this process activates detoxification routes which finally results in a reduction of the initial signaling molecules and explains how the HaCaT cells could reduce H_2_O_2_ concentrations already 1 h after blue light irradiation (Fig. 1).

While low concentrations of ROS act in a protective way^28^ high concentrations of ROS are well known to be able to irreversibly destroy cellular structures^28^. Although, gene expression analysis did not show any cell repair mechanisms, FACS analysis was used to test for apoptosis of the cells 24 h after 30 min of blue light irradiation. To exclude any cytotoxicity a separate assay would have to be performed, however, the cells did not show any signs of apoptosis (Fig. 2), which fits to the hypothesis that light-induced ROS concentrations are not too high and do not damage the cells.

Gene expression analysis revealed a high number of deregulated genes already one 1 h after irradiation, with even increasing numbers for 3 h and highest numbers 24 h after irradiation (Table 1 and Supplementary data 4). Subsequent GSEA depicted that blue light deregulates a variety of pathways in a time dependent manner (Fig. 4), with some pathways already deregulated 1 h after irradiation, which consequently induce the early response of blue light irradiation.

One of those early pathways is the pathway of metabolism of xenobiotics by CYPs (Fig. 4) with CYP1A1, which is also known as aryl hydrocarbon hydroxylase^29^, and CYP1B1 as highly upregulated genes (Fig. 7). They are best known for their metabolic activation of polycyclic aromatic hydrocarbons (PAHs) and heterocyclic aromatic amines/amides (HAAs) to electrophilic reactive intermediates^29^,^30^,^31^. Their gene expression is regulated by a heterodimeric transcription factor consisting of the aryl hydrocarbon receptor nuclear translocator (ARNT) and the aryl hydrocarbon receptor (AHR)^30^,^31^. The latter belongs to the group of basic helix-loop-helix (bHLH) PAS (homologous to Per/ARNT/Sim) proteins^32^ and is a ligand activated transcription factor usually defined as transcriptional regulator connected to adaptive xenobiotic response^33^. The ligand binding pocket of the AHR is able to fit a large number of planar, hydrophobic compounds^34^ with PAHs and HAAs as well-known exogenous ligands^35^. However, rising evidence led to the discovery of the existence of endogenous AHR ligands^35^ indicating that physiological functions of AHR are important for normal cell development and immune responses^33^,^36^.

AHR serves not only as an internal oxygen and redox status sensor, but also recognizes low molecular-weight compounds and light^37^,^38^ with endogenous ligands derived from tryptophan due to UV or visible light exposure induced photolytic destruction/photo-oxidation^32^,^35^,^39^. As the epidermis, consisting mainly of keratinocytes, has a high tryptophan content, the irradiation of keratinocytes with 453 nm blue light for 30 min respectively 41.4 J/cm^2^ may be able to induce the production of high affinity AHR ligands like 2,3,7,8-Tetrachlordibenzodioxin (TCDD), 6-formylindolo[3,2-b]carbazole (FICZ), 6,12-diformylindolo[3,2- b]carbazole (dFICZ) and oxi FICZ carboxylic acid type originating from indolo[3,2-b]carbazole-6-carboxylic acid (CICZ) which are natural substrates for CYPs present in skin cells^32^. After ligand binding AHR, which is located in the cytoplasm in its inactive state, forms a heterodimer with ARNT and translocates to the nucleus. Subsequently, it binds to the AHR-mediated aromatic hydrocarbon response element (AHRE, also XRE or DRE) DNA motif^29^, which leads to an upregulated transcription of a battery of xenobiotic-metabolizing enzymes (XMEs)^40^, which are collectively referred to as “AHR gene battery”^33^ (Fig. 5). These target genes are encoding phase I and phase II xenobiotic-metabolizing enzymes, which are vital for detoxification of xenobiotics^29^,^33^. The main enzymes encoded by AHR affected genes that are involved in phase I of xenobiotic metabolism are CYP1A1, CYP1A2, CYP1B1, NQO1 and ALDH3A1, whereas UGT1A and GSTA1 are connected to phase II^29^,^33^,^40^.

Gene expression analysis revealed an upregulation of CYP1A1, CYP1B1, ALDH3A1, NQO1 and UGT1A5 already 1 h after blue light irradiation, which is stable up to 3 h (Fig. 7). CYP1A1, CYP1B1, ALDH3A1 and NQO1 show an even higher upregulation in gene expression 24 h after irradiation, whereas UGT1A remains at the same level. This gene-regulation downstream of AHR activation strengthens the hypothesis that AHR is activated due to photo-oxidation of tryptophan after blue light irradiation. Moreover, activation of metabolism of xenobiotics by CYPs can lead to an activation of steroid hormone biosynthesis with NQO1 and ALDH3A1 likewise involved^22^,^41^,^42^ fitting to the findings of upregulated genes in the pathway of steroid hormone biosynthesis in gene expression analysis for all tested time points after blue light irradiation (Fig. 4).

Another overall consequence of AHR activated gene expression is generation of electrophilic reactive intermediates which induce reactive oxygenated metabolite (ROM)-mediated oxidative stress^29^. This triggers, besides the AHR dependent gene activation via AHRE, the additional Nrf2 dependent gene activation via the electrophile response element (EPRE, (also ARE) DNA motif^29^ resulting in expression of phase II detoxification enzymes^43^,^44^ thereby reducing oxidative stress^44^.

In its inactive state the transcription factor Nrf2 is bound to the substrate adaptor protein Kelch-like ECH-associated protein 1 (Keap1), which mediates the ubiquitination and subsequent proteasomal degradation of Nrf2 by a Cullin3-dependent E3 ubiquitin ligase complex^45^,^46^. After AHR induces ROM-mediated oxidative stress Keap1 is not able to bind to Nrf2 anymore as critical cysteine residues of the protein are oxidized thereby changing its conformation. Subsequently, the unbound Nrf2 is translocated to the nucleus activating gene expression via EPRE^46^. Target genes are partially consistent with AHR activated AHRE transcribed genes comprising inter alia ALDH3A1, NQO1 and UGT1A^47^. This is in agreement with the gene expression results (Fig. 7). Additionally, gene expression of Nrf2 itself is upregulated after blue light irradiation (Fig. 7) causing a higher level of Nrf2 transcription factor and more effective activation of the downstream process for reducing oxidative stress, which was shown before by Miao and colleagues in ref. 48. Furthermore, Keap1 can degrade inhibitor of kappa light polypeptide gene enhancer in B-cells kinase beta (IKKβ), which leads to an inhibition of activation of nuclear factor of kappa light polypeptide gene enhancer in B-cells 1 (NF-κB)^46^,^49^ and thereby to an anti-inflammatory response^50^. Moreover, AHR can directly interact with the transcription factor JunB to modulate skin immune responses, which was shown to play an important role in suppression of psoriatic lesions in keratinocytes^36^. This can be observed in gene expression analysis where, besides the NF-κB signaling pathway, above all inflammatory pathways are downregulated (Fig. 4).

Cell cycle arrest pathway can be directly activated through ROS production^22^,^51^, however, AHR activation can influence the cell cycle, too^31^. After binding retinoblastoma 1 (RB1)^52^, the AHR-RBI complex can block E2F-mediated transcription of S phase genes like e.g. CDKN1B^31^,^53^,^54^, resulting in an inhibition of normal progression of G1 to S phase in cell cycle^31^. CDKN1B is downregulated 1 h and 3 h after blue light irradiation (Fig. 7), additionally to ROS induced cell cycle arrest. This explains the decrease of cell proliferation with blue light. However, at the time point 24 h after blue light irradiation CDKN1B is slightly downregulated. These findings fit to the gene expression results of the pathway of DNA replication, which was downregulated for 1 h and 3 h after irradiation but slightly upregulated 24 h after irradiation (Fig. 4).

Next to its function as transcription factor the AHR-ligand complex can associate with cell division cycle 37 control protein (Cdc37) and the non-receptor tyrosine kinase Src causing the dissociation of the latter. Consequently, Src translocation into the cell membrane is promoted where it phosphorylates the epidermal growth factor receptor (EGFR, also ERBB), which activates ERK1/2 (also MAPK3/1) target gene expression leading to cell survival^33^,^55^. The crucial time point for the cell to decide between cell survival and apoptosis after blue light irradiation seems to be during the first hour after irradiation. Here, oxidative stress is induced, which was described with an increase of H_2_O_2_ production 30min after blue light irradiation followed by a decrease already 1 h after irradiation. An early upregulation of ERK1/2 occurs 1 h after irradiation (Fig. 7) on gene expression level triggering cell survival pathways. This cell survival effect is emphasized by the additional downregulation of TFN signaling pathway (Fig. 4) containing TRADD, which can signal apoptosis^29^,^33^. TRADD is downregulated for all tested time points after blue light irradiation with a maximum after 3 h (Fig. 7).

Finally, AHR activation triggers the induction of AHRR gene expression 3 h after blue light irradiation (Fig. 7) which is known to lead to a dimerization of AHRR with ANRT and results in an inhibition of AHR function. Therefore, AHRR activation by AHR represents a regulatory biofeedback loop in the xenobiotic signal transduction pathway^40^,^56^,^57^.

qPCR was used to verify gene expression results of some selected genes. All selected genes showed the same deregulation in qPCR and gene expression analysis with CYP1A1, CYP1B1, ALDH3A1, NQO1 and UGT1A significantly upregulated and FOS, IL8 and Krt5 significantly downregulated for the time point 24 h after blue light irradiation.

Although gene expression results indicate AHR as a possible target, it should be noted that the direct, causal correlation of AHR to be a direct target of blue light treatment has to be further tested using for example gain or loss of function studies and/or protein levels of the named genes should be tested at the different time points to confirm the hypothesis. Furthermore, functional analysis should be performed to confirm the role of ROS and its connection to AHR using for example antioxidants.

## Conclusion

Gene expression evaluation of HaCaT cells revealed an upregulation of “AHR battery genes” leading to production of phase I and phase II enzymes of xenobiotic metabolism^29^,^33^. One important action of this downstream process is to provide a delicate hormesis between promoting and preventing ROM-mediated oxidative stress, which is in agreement with our ROS measurements. H_2_O_2_ concentrations are increased 30 min after blue light irradiation; however, already 1 h after irradiation H_2_O_2_ is metabolized by the cells leading to an even lower ROS concentration. Furthermore, steroid hormone biosynthesis is activated as a downstream process of “AHR battery gene” expression^19^,^41^,^42^,^51^ already 1 h after irradiation triggering anti-inflammatory responses^41^,^58^,^59^. Additionally, inflammation is also decreased due to oxidative stress inhibited NF-κB signaling pathway^46^,^49^,^50^ and interaction with JunB^36^. DNA replication pathway is downregulated resulting in a decrease in cell proliferation due to primary production of ROS^51^ and AHR-induced downregulation of CDKN1B^31^. However, ROS concentrations are not reaching a damaging level as cell survival pathways are enhanced by crosstalk of AHR-ligand complex with EGFR. Moreover, reduction of TNF-signaling pathway and downregulation of TRADD gene expression, which are relevant for apoptotic signaling, are consistent with FACS analysis as 24 h after blue light irradiation cells are not showing any sign of apoptosis. Finally, it can be concluded that gene expression shows a time course after blue light irradiation, with early response genes and pathways leading to the identification of AHR as a possible target for PBM with blue light via photo-oxidation of tryptophan resulting, when using this described dose, in a cell protective effect with decreased proliferation, production of steroid hormones and prevention of inflammatory responses.

## Methods

### Cell culture

HaCaT cells (immortal human keratinocytes) from Cell Line Service (CLS) GmbH (Heidelberg/Germany) were cultured as previously described^22^ under standard conditions at 37 °C with 5% CO_2_. They were cultured in Dulbecco’s modified Eagle’s medium (DMEM) high glucose containing 10% fetal bovine serum (FBS), 1mM sodium pyruvate and 100 U/mL penicillin/streptomycin (Gibco^®^ by life technologies TM AG (Carlsbad/USA)) whereas 0.1% Trypsin-EDTA (1x) phenol red from Gibco^®^ was used to detach the cells. Subculturing ratios have been 2/10 to 3/10.

### Blue light irradiation

HaCaT cells per well were plated in black 96 well plates, with sterile clear flat bottom wells (Sigma Aldrich Co. LLC, St. Louis/USA)^22^. After seeding, cells were incubated 24 h at 37 °C with 5% CO_2_. Medium was renewed and cells were illuminated for 30 min. The right half of the plate was taped with black foil for the no light negative control. After defined time points cells were harvested with TRIzol Reagent (Ambion^®^ by life technologies TM AG (Carlsbad/USA)) and stored at −80 °C for further use in RNA isolation and following gene expression analysis with microarrays. Experiments were conducted in triplicates and repeated twice.

Lumileds LUXEON Rebel LXML-PR01-0275 were used (Koninklijke Philips N.V., Eindhoven/Netherlands) with an treatment surface irradiance of 23 mW/cm^2^ at an irradiation distance of 55 mm, beam divergence was ±15° and a peak wavelength of 453 nm (blue light).

### ROS measurement

Amplex UltraRed (Moelcular Probes, Invitrogen, Carlsbad, CA) was used for measuring H_2_O_2_ concentrations in HaCaT cells^21^. At defined time points after 30 min of blue light irradiation 50 μl 0,1 M Potassium phosphate buffer pH 6,0 containing 100 mM Amplex Ultrared and 0.2 U/ml Horse radish peroxidase (Molecular Probes, Invtirogen, Carlsbad, CA) was added to each well and incubated for 20 min at 37 °C with 5% CO_2_. Fluorescence was measured with the Infinit^®^ 200 PRO microplate reader from underneath at λ_ex_490 nm/λ_em_581 nm (Tecan Group AG, Männedorf/Switzerland). Experiments were conducted in triplicates and repeated twice.

### FACS

For analyzing apoptosis cells were labeled with FITC Annexin V (BioLegend, San Diego/USA) and Propidiumiodide (PI) (InvitrogenTM by life technologies TM AG (Carlsbad/USA)) supernatant was harvested to collect possible apoptotic cells. After that, cells were washed with PBS, trypsinized and dissolved with the collected supernatant; 2 × 10^5^cells were used. The cells were transferred to a 15 ml Falcon tube and centrifuged 3 min at RT and 2000 × g. The supernatant was removed and the pellet was washed twice, first with PBS, secondly with Annexin-Binding Buffer (BioLegend(San Diego, USA)). Subsequently, the pellet was resuspended in 100 μl Annexin-Binding Buffer and cells were incubated with 5 μl of Annexin V, and 2 μl of PI 1 mg/ml for 15 min at RT in the dark. Finally, 100 μl Annexin-Binding Buffer was added. For positive control 1 μM Staurosporine (Sigma Aldrich Co. LLC, St. Louis/USA) was added for 4 h to HaCaT cells to induce apoptosis. The subsequent measurement was performed on a BD FACSCalibur (BD Biosciences, Heidelberg, Germany) while Flowing Software version 2.5.1 was used to perform a distribution analysis for statistical evaluation. Experiments were conducted in triplicates and repeated twice.

### RNA isolation for microarray analysis and quantitative real time PCR

RNA was isolated as described in the TRIzol Reagent protocol. The RNA pellet was re-suspended in 20 μl RNase-free water.

### Gene expression analysis with Affymetrix GeneChips

After RNA isolation RNA was purified^22^ using the RNA Clean-Up and Concentration Micro Kit. cDNA synthesis was performed using the SuperScript Choice System according to the recommendations of the manufacturer. Using ENZO BioArray HighYield RNA Transcript Labeling Kit biotin-labeled cRNA was produced. Standard protocol from Affymetrix was used for the *in vitro* transcription (IVT). Quantification of cRNA was performed by spectrophotometric analysis with acceptable A260/A280 ratio of 1.9 to 2.1. Fragmentation of the cRNA was achieved using the Affymetrix protocol. For gene expression profiling, labeled and fragmented cRNA was hybridized to Affymetrix Hugene-2_0st microarrays with an incubation of 16 h at 45 °C. The Affymetrix fluidics station 450 was used to wash the microarrays, scanning was performed with Affymetrix GeneChip scanner 3000.

### Bioinformatics analysis-Affymetrix GeneChips

The Custom CDF Version 18 with Entrez based gene definitions was used for annotation^60^. Applying quantile normalization, the raw fluorescence intensity values were normalized. To remove the plate effect, a batch normalization based on k-means was performed. Based on OneWay-ANOVA, differential gene expression was analyzed using a commercial software package: SAS JMP10 Genomics, version 6, from SAS. A false positive rate of a = 0.05 with FDR correction was taken as the level of significance.

Gene Set Enrichment Analysis (GSEA) was performed to determine whether defined lists (or sets) of genes exhibit a statistically significant bias in their distribution within a ranked gene list (see http://www.broadinstitute.org/gsea/ for details (Subramanian *et al*., 2005)). Pathways belonging to various cell functions such as cell cycle or apoptosis were obtained from the public database KEGG. As KEGG does not contain an AHR signaling pathway, Aryl Hydrocarbon Receptor Pathway (Homo sapiens) from Rianne Fijten, Egon Willighagen, Alexander Pico, *et al*. was used (http://wikipathways.org/index.php/Pathway:WP2873) for statistical analysis.

The raw and normalized data are deposited in the Gene Expression Omnibus database (http://www.ncbi.nlm.nih.gov/geo/; accession No. GSE82094).

### Reverse Transcription PCR

1 μg of previously isolated RNA was used for the preparation of cDNA. RNA was filled up with distilled water to a total volume of 11 μl. A master mix was prepared according to RevertAid H Minus First Strand cDNA Synthese Kit from Thermo Fisher Scientific Inc. (Waltham/USA). The 20 μl reaction mixture was then used with the following program for the production of cDNA: 5 min at 25 °C, 60 min at 42 °C and 5 min at 70 °C. The cDNA was 1:10 diluted for further use in Real Time-PCR.

### Primer design

Primer sequences for CYP1A1, CYP1B1, ALDH3A, NQO1, and UGT1A were adopted from Brauze *et al*.^40^. Primers for FOS, IL8, Krt5 and MOK (used as reference gene) were designed according to published genes sequences (NCBI-Gene) with PrimerBlast (http://www.ncbi.nlm.nih.gov/tools/primer-blast/) and span exon/exon boundaries Table 2. BLAST alignment search (http://blast.ncbi.nlm.nih.gov/Blast.cgi) was used to verify specificity. All Primers were purchased as DNA Oligo-Primer from Metabion International AG (Planegg, Steinkirchen/Germany).

### Real-time PCR

To verify expression of various genes in the microarray analysis, a Real Time-PCR (qPCR) was carried out with SYBR Green. The reaction mixture consisted of 5 μl 1:10 diluted cDNA, 4.8 μl water, 10 μl SYBR Green master mix and 0.1 μl each primer (LightCycler 96 DNA Green, Roche Diagnostics GmbH (Mannheim/Germany)). DNA Oligo-Primer from Metabion International AG (Planegg, Steinkirchen/Germany) were used with a concentration of 100 μM, therefore end concentration in the reaction mix was 0.5 μM. The qPCR was programmed as follows: 10 min 95 °C, 45x(10 sec 95 °C, 10 sec at Primer specific Tm, 10 sec 72 °C), 10 sec 95 °C, 1 min 65 °C, 1 sec 97 °C. Experiments were conducted in triplicates and repeated twice.

### Bioinformatics analysis-qPCR

For cPCR evaluation Roche LightCycler^®^ 96 Application Software and SAS JMP10 Genomics, version 6, were used.

## Additional Information

**How to cite this article**: Becker, A. *et al*. Gene expression profiling reveals aryl hydrocarbon receptor as a possible target for photobiomodulation when using blue light. *Sci. Rep*. **6**, 33847; doi: 10.1038/srep33847 (2016).

## Supplementary Material

Supplementary Information

Supplementary Table S1

## Figures and Tables

**Table 1:** Significantly deregulated genes and GSEA (Irradiation time in minutes, harvesting time in h).

Irradiation time	30 min	30 min	30 min
Harvesting time	1 h	3 h	24 h
Significant differentially expressed genes	1358	1686	2192
Significant upregulated genes	656	885	1090
Pathways containing upregulated genes	105	105	119
Significant pathways containing upregulated genes with FDR <25%	0	10	21
Significant pathways containing upregulated genes with nominal p-value < 5%	6	17	23
Significant downregulated genes	702	801	1102
Pathways containing downregulated genes	153	152	138
Significant pathways containing downregulated genes with FDR < 25%	0	6	0
Significant pathways containing downregulated genes with nominal p-value < 5%	5	17	13

**Table 2:** Primer specifications.

Gene name and Accession number	Sequence	Annealing Temperature Tm(°C)/t(s)	Efficiency	R2
FOS	F: CACTCCAAGCGGAGACAGAC	63	1.92	0.96
NM_005252.3	R: AGGTCATCAGGGATCTTGCAG	61		
IL8	F: AGGAACCATCTCACTGTGTGT	59	2.14	0.97
NM_ 000584	R: CACCCAGTTTTCCTTGGGGTC	63		
Krt5	F: AACCTGGACCTGGATAGCATCA	62	1.80	0.78
NM_ 000424.3	R: ACATTGTCAATCTCGGCTCTCAG	63		
MOK	F: AGAGATCCAAGCACTGAGGC	60	2.12	0.98
NM_ 001272011.1	R: TACCAGCGGGTGGAGATGTA	60		
