# Supplementary material for: Gene expression profiling reveals aryl hydrocarbon receptor as a possible target for photobiomodulation when using blue light
Source: Sci Rep. 2016 Sep 27;6:33847. doi: 10.1038/srep33847 (PMC5037386; doi:10.1038/srep33847)
Supplement: Supplementary Information [file srep33847-s1.doc]

# Gene expression profiling reveals aryl hydrocarbon receptor as a possible target for photobiomodulation when using blue light

Anja Becker^1^, Anna Klapczynski^1^, Natalia Kuch^1^, Fabiola Arpino^1^, Katja Simon-Keller^1^, Carolina De La Torre^1^, Carsten Sticht^1^, Frank A. van Abeelen^2^, Gerrit Oversluizen^2^, Norbert Gretz^1^

^1^Medical Research Centre, University of Heidelberg, D-68167 Mannheim, Germany

^2^Philips Group Innovation, Research, High Tech Campus 34, 7.031, 5656 AE Eindhoven, The Netherlands

Supplementary data 1: Gene expression analysis - volcano plot 1h after blue light irradiation.

Supplementary data 2: Gene expression analysis - volcano plot 3h after blue light irradiation.

Supplementary data 3: qPCR of selected genes verifies gene expression analysis results. Fold changes are significant for all genes.
